# Impact of Short-Term Exposure to Nitrogen Dioxide (NO_2_) and Ozone (O_3_) on Hospital Admissions for Non-ST-Segment Elevation Acute Coronary Syndrome

**DOI:** 10.3390/toxics12020123

**Published:** 2024-02-01

**Authors:** Andreea-Alexandra Rus, Silvius-Alexandru Pescariu, Adrian-Sebastian Zus, Dan Gaiţă, Cristian Mornoş

**Affiliations:** 1 Cardiology Department, “Victor Babes” University of Medicine and Pharmacy, 2 Eftimie Murgu Sq., 300041 Timisoara, Romania; pescariu.alexandru@umft.ro (S.-A.P.); adrian.zus@umft.ro (A.-S.Z.); dgaita@cardiologie.ro (D.G.); mornos.cristian@umft.ro (C.M.); 2 Research Center of the Institute of Cardiovascular Diseases Timisoara, 13A Gheorghe Adam Street, 300310 Timisoara, Romania; 3 Institute of Cardiovascular Diseases Timisoara, 13A Gheorghe Adam Street, 300310 Timisoara, Romania

**Keywords:** non-ST-segment elevation acute coronary syndrome, short-term exposure, air pollution, environmental health, environmental risk factors

## Abstract

In the context of recent climate change, global warming, industrial growth, and population expansion, air pollution has emerged as a significant environmental and human health risk. This study employed a multivariable Poisson regression analysis to examine the association between short-term exposure to atmospheric pollutants (nitrogen dioxide—NO_2_, sulfur dioxide -SO_2_, ozone—O_3_, and particulate matter with a diameter less than 10 μm-PM_10_) and hospital admissions for non-ST-segment elevation acute coronary syndrome (NSTE-ACS). Daily data on NSTE-ACS admissions, air pollutants, and meteorological variables were collected from January 2019 to December 2021. Elevated NO_2_ concentrations were associated with a higher risk of NSTE-ACS hospitalization, notably in spring (OR: 1.426; 95% CI: 1.196–1.701). Hypertensive individuals (OR: 1.101; 95% CI: 1.007–1.204) and those diagnosed with unstable angina (OR: 1.107; 95%CI: 1.010–1.213) exhibited heightened susceptibility to elevated NO_2_ concentrations. A 10 μg/m^3^ increase in NO_2_ during spring at lag 07 (OR: 1.013; 95% CI: 1.001–1.025) and O_3_ in winter at lag 05 (OR: 1.007; 95% CI: 1.001–1.014) was correlated with an elevated daily occurrence of NSTE-ACS admissions. Short-term exposure to various air pollutants posed an increased risk of NSTE-ACS hospitalization, with heightened sensitivity observed in hypertensive patients and those with unstable angina. Addressing emerging environmental risk factors is crucial to mitigate substantial impacts on human health and the environment.

## 1. Introduction

In recent years, marked by shifts in climate patterns, the rise in global temperatures, expansions in industry, and population surges, air pollution has emerged as a substantial threat to both the environment and human well-being. The World Health Organization (WHO) underscores the gravity of outdoor air pollution, attributing about 4.2 million premature deaths annually to its effects, with 37% linked to stroke and cardiovascular diseases [1]. Additionally, WHO’s 2020 report highlighted an estimated 3.2 million deaths yearly due to household air pollution, with nearly a third of the global populace still resorting to inadequate methods for household heating and cooking, such as burning coal, wood, and crop residues [2]. The primary culprits behind external environmental pollution encompass agricultural practices, industrial activities, and transportation, whereas indoor pollutants predominantly stem from household fuel combustion [3]. The European Society of Cardiology (ESC) in 2019 advised individuals with a history of cardiovascular diseases (CVD) to steer clear of congested traffic zones, use protective masks in densely populated areas, and deploy air filters to alleviate indoor air pollution [4]. Alarmingly, it has been estimated that roughly three billion cases of health issues related to pollution afflict individuals, particularly those with precarious socio-economic statuses [5].

Outdoor air pollution is a complex combination of particulate matter (PM_10_—particulate matter with a diameter ≤10 μm; PM_2.5_—particulate matter with a diameter ≤2.5 μm; UFP—particulate matter with a diameter ≤ 0.1 μm) and gaseous pollutants (NO_2_—nitrogen dioxide; O_3_—ozone; SO_2_—sulfur dioxide; CO—carbon monoxide) originating from diverse emission sources. PM comprises both directly emitted particles, such as diesel soot, and secondary particles formed through physicochemical processes involving gases. Examples of secondary particle formation include the creation of nitrates and sulfates from gaseous nitric acid and SO_2_, respectively. The release of PM into the atmosphere stems from various sources, including natural processes, fuel combustion, transportation systems, construction and demolition activities, residential wood burning, agricultural practices, airborne pollens and molds, windblown soil, as well as events like forest fires and the combustion of agricultural debris. O_3_ is a highly reactive gas that appears colorless to bluish and possesses a distinctive odor associated with electrical discharges. It has been acknowledged as the primary component of photochemical smog since the 1950s. In the troposphere, O_3_ is produced through the interaction of solar ultraviolet (UV) radiation with nitrogen oxides and reactive hydrocarbons emitted by various sources, including motor vehicles and industrial facilities. This process involves the photolysis of NO_2_ into nitrogen monoxide (NO) and oxygen atoms. The oxygen atoms subsequently react with molecular oxygen to create O_3_. The photochemical formation of O_3_ is particularly prominent on warm, sunny days, primarily due to industrial and transportation-related activities. CO is a gas that lacks odor, color, and taste. It exhibits a binding affinity to hemoglobin that is 250 times higher than that of oxygen, consequently impeding the effective delivery of oxygen to body tissues. CO is produced through the incomplete combustion of carbonaceous fuels such as natural gas, wood, charcoal, and oil. Under specific conditions, such as inadequately ventilated parking structures, concentrations of CO may rise to levels capable of causing substantial increases in carboxyhemoglobin. This is particularly relevant for individuals with notable atherosclerotic disease or other cardiac conditions. SO_2_ is a colorless, soluble gas with a distinctive strong odor and taste, causing considerable irritation. SO_2_ is generated by the smelting of mineral ores and the combustion of oil and coal. In settings outside of occupational environments, SO_2_ is typically present at significantly lower concentrations indoors compared to outdoor levels. Nevertheless, the utilization of kerosene space heaters has the potential to elevate indoor concentrations significantly. Fuel combustion in the industrial and transportation sectors stands out as the predominant contributor to NO_2_ emissions. The production of tropospheric ozone and other photochemical oxidants begins through the process of NO_2_ photolysis. At the same time, NO functions as a scavenger, mitigating the levels of ozone in the atmosphere [1,5,6].

Acute coronary syndrome (ACS) serves as the clinical presentation of coronary artery disease and encompasses two distinct categories: ST-segment elevation myocardial infarction (STEMI) and non-ST-segment elevation acute coronary syndrome (NSTE-ACS). NSTE-ACS is further classified into two principal subtypes, namely, non-ST-segment elevation myocardial infarction (NSTEMI) and unstable angina (UA). It is important to note that the underlying mechanisms of STEMI and NSTE-ACS differ. STEMI arises following complete vessel occlusion due to a ruptured atheroma plaque accompanied by thrombosis. In contrast, NSTEMI is characterized by partial occlusion of coronary arteries or an imbalance between myocardial oxygen demand and supply. The pathogenic mechanisms of UA and NSTEMI are similar, with the distinction that UA lacks evidence of myocardial damage based on cardiac biomarkers [7].

Recently, there has been a growing emphasis on conducting epidemiological and clinical research aimed at uncovering new risk factors. These studies strive to pinpoint and assess the harmful impacts of ambient air pollution on cardiovascular health and mortality. The earliest signs of a link between industrial smog and premature deaths caused by cardio-respiratory diseases trace back to the early 20th century [8,9]. Subsequent investigations consistently highlight that exposure to air pollutants, whether over short or long periods, significantly contributes to negative outcomes in cardio-respiratory conditions [10,11,12,13,14,15,16,17]. Earlier epidemiological discoveries specifically emphasize the heightened detrimental effects of PM on CVD compared to other airborne pollutants, especially in cases of increased exposure to PM_2.5_ [10,13,14,15,16,17,18,19,20].

Considering research findings that have revealed detrimental effects of pollutants at levels considerably lower than those outlined by established guidelines, the WHO has significantly adjusted the reference values for most atmospheric pollutants, including both annual and 24-h average values [1,21,22]. The updated WHO guide now advocates maximum 24-h reference values as follows: 25 μg/m^3^ for NO_2_; 45 μg/m^3^ for PM_10_; 15 μg/m^3^ for PM_2.5_; 40 μg/m^3^ for SO_2_; and 4 mg/m^3^ for CO. Moreover, it sets the average of the daily maximum 8-hour mean concentration for O_3_ at 100 μg/m^3^ [1].

Researchers have noted that inhaling elevated concentrations of gaseous pollutants and PM induces prothrombotic and proinflammatory effects, endothelial dysfunction, and an elevated lipid load due to heightened oxidative stress. These alterations collectively contribute to the initiation and progression of atherosclerotic plaques [23,24,25,26]. Despite numerous in vitro and in vivo studies, a complete understanding of the pathophysiological mechanisms underlying the increased risk of ACS due to air pollution exposure remains elusive. While many investigations have looked at how pollution affects respiratory and cardiovascular diseases, not many studies have focused on how it might lead to hospitalization for NSTE-ACS. There is not enough information on this specific heart condition, showing a gap in our knowledge. This highlights the need for more research in this area because there is not much data available in current studies. This study endeavors to evaluate the correlation between short-term exposure to atmospheric pollutants, namely, NO_2_, SO_2_, O_3_, and PM_10_, and hospital admissions for NSTE-ACS. We conducted comprehensive analyses encompassing both an overall assessment and a subgroup breakdown based on seasonal variations, gender disparities, age groups, cardiovascular risk factors, coronary artery disease (CAD), and the specific type of NSTE-ACS. Furthermore, we explored potential interactions with meteorological factors. Unraveling the adverse influence of ambient air pollution on the incidence of acute coronary events holds the potential to offer valuable insights crucial for devising effective policies aimed at mitigating pollutant levels. Consequently, this could lead to a reduction in associated health risks.

## 2. Materials and Methods

### 2.1. Hospital Admission Data

This prospective observational study was conducted at the University Hospital of Timisoara, Romania, encompassing patients hospitalized for NSTE-ACS from January 2019 to December 2021. The diagnosis of NSTE-ACS was established through clinical criteria (persistent precordial pain lasting at least 20 min or anginal episodes of approximately 15 min) and paraclinical indicators (persistent depression of the ST segment by at least 1 mm on electrocardiogram, segmental abnormalities of myocardial wall movements on transthoracic echocardiography, and high-sensitivity troponin I within normal limits for UA or increased and/or decreased serum levels of myocardial necrosis enzymes for NSTEMI). The analysis incorporated clinical and demographic characteristics, patients’ home addresses, NSTE-ACS type (NSTEMI and UA), and the history of comorbidities (hypertension, diabetes mellitus, and dyslipidemia).

Patients under the age of 18, individuals diagnosed with STEMI, chronic coronary syndrome (CCS), or other cardiovascular diseases, and those who did not reside in the study geographic area shortly before hospitalization were excluded from the data collection process. All patients included in the analysis provided written informed consent. Additionally, this study adheres to the principles outlined in the Declaration of Helsinki and has received approval from the Research and Development Ethics Committee of the Institute of Cardiovascular Diseases in Timisoara, Romania (Approval No. 1658/28.03.2014).

### 2.2. Air Pollution and Climatological Data

Romania features a temperate-continental climate marked by four distinct seasons: winter (December–February); spring (March–May); summer (June–August), and fall (September–November). The geographic study area is characterized by hot and dry summers, where air temperatures can exceed 30 °C, and winters with low atmospheric temperatures, reaching as low as −20 °C [27]. Patients admitted for NSTE-ACS are from five regions in the southwest area of the country, each exhibiting specific variations in terms of air pollution levels and daily average meteorological parameters.

Atmospheric pollutants were sourced from the Romanian National Institute for Research and Development for Environmental Protection (INCDPM). Data were collected from 24 fixed monitoring stations, with the selection of the pollution monitoring device situated in the closest proximity to the patient’s residence. This paper included the daily average concentrations of NO_2_, O_3_, SO_2_, and PM_10_. Notably, PM_2.5_ data were excluded from the analysis due to many missing values during the study period. Details about air pollutant concentrations are publicly accessible online (https://www.calitateaer.ro, accessed on 4 March 2023).

The meteorological variables considered in this study were the daily averages of air temperature and relative humidity. These values were provided by the Romanian National Meteorological Association and collected from weather stations near the patient’s residence. All meteorological and pollution data were recorded as average daily values spanning from the day of hospitalization (lag 0) to seven days preceding the onset of the acute coronary event (lag 7).

### 2.3. Statistical Analysis

We employed a Generalized Linear Model (GLM) with a Poisson distribution and a Distributed Lag Model (DLM) covering a week. This approach aimed to explore the connection between short-term exposure to air pollutants and hospital admissions for NSTE-ACS. The choice of these statistical methods is justified by the Poisson-type distribution observed in the daily count of NSTE-ACS hospitalizations. A multivariable Poisson regression analysis model was developed, incorporating meteorological factors and concentrations of ambient air pollutants. Considering the coexistence of various environmental pollutants, we adopted a comprehensive approach by integrating all air pollutants into the model. Our investigation focused on the impact of atmospheric pollutants exceeding reference levels established by the WHO guide on daily hospitalizations for NSTE-ACS at lag 01 (“lag 01” represents the moving average of the current day and the previous day’s exposure to air pollutants) [12]. Additionally, pollutants like O_3_ and SO_2_, surpassing predefined thresholds on a limited number of days (0.52% for O_3_ and 0.37% for SO_2_), were included for a more accurate evaluation of atmospheric pollutants’ influence on hospitalization rates for acute coronary events. Subsequently, we conducted an analysis for each increment of ≥10 μg/m^3^ in environmental pollutants at various time lags (e.g., lags 03, 05, and 07) concerning NSTE-ACS incidents [28,29]. Including a time lag is essential, as it allows for the observation of the impact of pollutant exposure not only on the day of exposure but also over subsequent days. The designations “lag 03”, “lag 05” and “lag 07” represented the moving averages of air pollutant concentrations from the previous three, five, and seven days, respectively.

We conducted a comprehensive analysis, as well as a stratified analysis based on seasons (winter: December–February; spring: March–May; summer: June–August; autumn: September–November), type of NSTE-ACS (NSTEMI and UA), and individual characteristics, such as gender (male and female), age (<65 years and ≥65 years), CAD (single-vessel lesions and multi-vessel lesions), and cardiovascular risk factors (patients with and without hypertension and patients with and without diabetes mellitus). The objective was to investigate whether the effect estimates varied across these variables. The findings were presented as odds ratio (OR), along with 95% confidence intervals (CI). Additionally, we summarized the correlations between daily concentrations of air pollutants and daily meteorological factors using Spearman’s correlation.

The Kolmogorov–Smirnov test assessed the distribution of variables. Continuous variables were presented as mean values with standard deviation (SD), while categorical variables were expressed as numbers and percentages. Comparative analysis of numerical data employed the Independent Samples *t*-test, and the Pearson Chi-Squared Test was used for categorical variables. All analyses were conducted using IBM SPSS Version 26.0 Software, and graphical representations were created using Excel Version 2019. A significance threshold of *p* < 0.05 was set for all tests.

## 3. Results

### 3.1. Characteristics of Patients Hospitalized for Non-ST-Segment Elevation Acute Coronary Syndrome

This study encompassed 1547 patients admitted for NSTE-ACS, among whom 264 individuals (17.1%) received a diagnosis of NSTEMI, while 1283 (82.9%) were hospitalized for UA. The average age of the patients stood at 63.53 ± 10.0, with 48.5% of participants aged 65 years or older. Men constituted the majority, representing 72.5% of the study cohort. In terms of cardiovascular risk factors (CVRF), a significant proportion of hospitalized patients had a history of hypertension (HTN) (86.7%), while smaller percentages had a history of diabetes mellitus (DM) (32.7%) and hypercholesterolemia (38%). Primary percutaneous coronary intervention (PCI) emerged as the main therapeutic approach (84.4%), with 71.8% of patients presenting multi-vessel coronary artery disease. The clinical and paraclinical characteristics of the study population are thoroughly outlined in Table 1.

**Figure 1:**
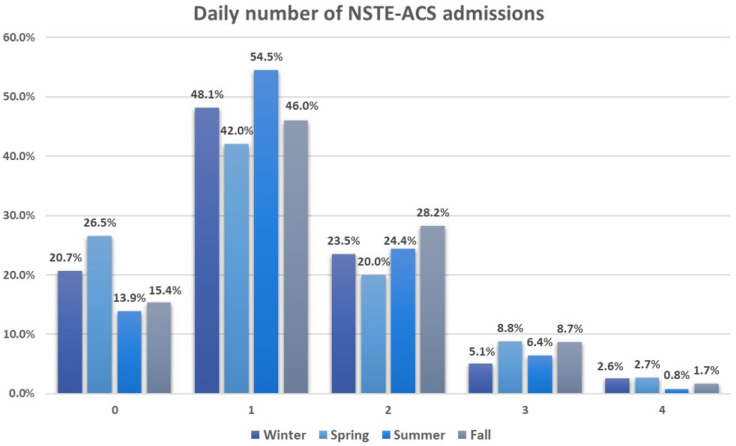
Daily number of non-ST-segment elevation acute coronary syndrome admissions across seasons. All values are expressed as percentages. 0—indicates zero hospitalizations per day (with a total of 361 days); 1—signifies one hospitalization per day (with a total of 913 days); 2—denotes two hospitalizations per day (with a total of 460 days); 3—represents three hospitalizations per day (with a total of 138 days); 4—indicates four hospitalizations per day (with a total of 36 days).

The average daily hospitalization rate throughout the study duration stood at 1.25 ± 0.91. Around 47.9% of the days recorded a single hospitalization, whereas only 9.1% of days saw three or four hospitalizations. Seasonal breakdown revealed an increased frequency of three to four hospitalizations per day during spring, whereas summer predominantly experienced one hospitalization per day, accounting for 54.5%. Figure 1 illustrates the daily count of NSTE-ACS admissions across different seasons.

### 3.2. Daily Air Pollutants and Meteorological Variables Features

Throughout the entire study duration, the mean daily concentration of NO_2_ averaged at 23.0 ± 11.7 μg/m^3^, while O_3_ levels registered at 43.9 ± 19.3 μg/m^3^ daily. SO_2_ exhibited a mean concentration of 10.2 ± 3.6 μg/m^3^, and PM_10_ showed an average concentration of 21.2 ± 13.6 μg/m^3^. Additionally, the average daily values for air temperature and relative humidity were 12.7 ± 8.5 °C and 72.4 ± 14.2%, respectively. Detailed information regarding means, standard deviations (SD), minimum and maximum values, as well as selected percentiles of pollutants and meteorological variables, can be found in Table 2.

**Figure 2:**
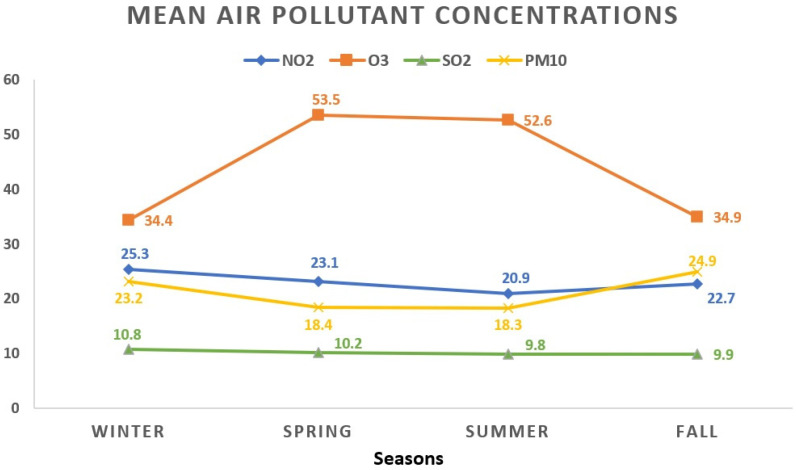
The seasonal variations in mean air pollutant concentrations. All values represent the mean pollutant concentration depending on the seasons and are expressed as μg/m^3^. The total of 1908 data days does not correspond to the total calendar days over the 3-year period due to data collection from 5 regions, each with specific variations in daily air pollution levels: winter (468 days); spring (441 days); summer (516 days); fall (483 days). NO_2_: nitrogen dioxide; O_3_: ozone; SO_2_: sulfur dioxide; PM_10_: particulate matter with a diameter ≤10 μm.

During the winter season, NO_2_ showed the highest mean concentration at 25.3 ± 13.2 μg/m^3^, contrasting with the lowest levels observed during summer at 20.9 ± 10.6 μg/m^3^. O_3_ exhibited its peak average value in spring (53.5 ± 17.8 μg/m^3^), while the lowest readings were noted in winter days (34.4 ± 17.3 μg/m^3^). The winter period displayed the highest average concentration for SO_2_ (10.8 ± 3.9 μg/m^3^), whereas the summer season recorded the lowest (9.8 ± 3.6 μg/m^3^). As for PM_10_, the highest average level appeared in autumn (24.9 ± 17.5 μg/m^3^), while the lowest occurred during summer (18.3 ± 9.1 μg/m^3^). Figure 2 visually represents the seasonal fluctuations in mean air pollutant concentrations.

**Figure 3:**
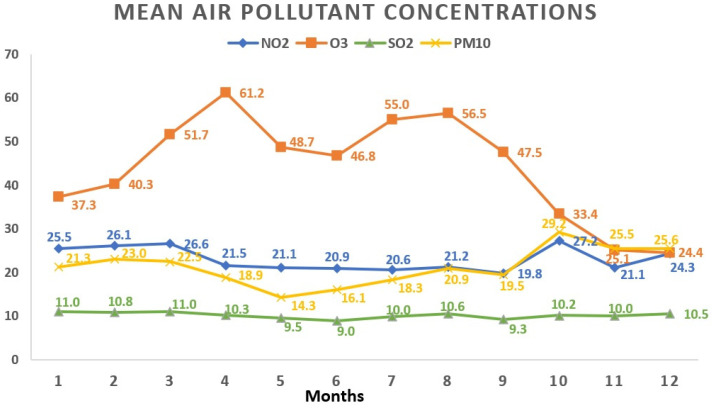
Variations in mean air pollutant concentrations across various months. All values represent the mean pollutant concentration depending on the months and are expressed as μg/m^3^. The total of 1908 data days does not correspond to the total calendar days over the 3-year period due to data collection from 5 regions, each with specific variations in daily air pollution levels: January (165 days); February (161 days); March (147 days); April (135 days); May (159 days); June (181 days); July (172 days); August (163 days); September (151 days); October (162 days); November (170 days); December (142 days). NO_2_: nitrogen dioxide; O_3_: ozone; SO_2_: sulfur dioxide; PM_10_: particulate matter with a diameter ≤10 μm.

**Figure 4:**
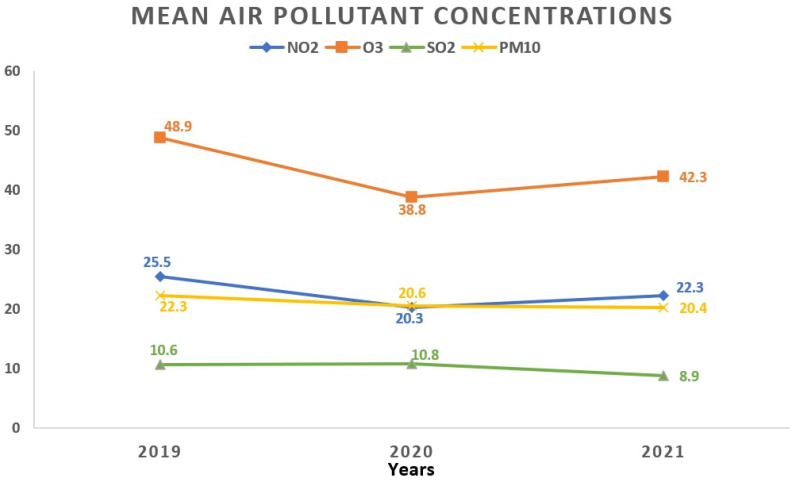
Temporal fluctuations in average air pollutant concentrations over various years. All values represent the mean pollutant concentration depending on the years and are expressed as μg/m^3^. The total of 1908 data days does not correspond to the total calendar days over the 3-year period due to data collection from 5 regions, each with specific variations in daily air pollution levels: 2019 (760 days); 2020 (571 days); 2021 (577 days). NO_2_: nitrogen dioxide; O_3_: ozone; SO_2_: sulfur dioxide; PM_10_: particulate matter with a diameter ≤10 μm.

Regarding the monthly breakdown, the greatest average concentrations for NO_2_ and PM_10_ surfaced in October (27.2 ± 15.1 μg/m^3^ and 29.2 ± 24.1 μg/m^3^, respectively). SO_2_ reached its apex in March (11.0 ± 3.7 μg/m^3^), while O_3_ peaked in April with an average concentration of 61.2 ± 17.4 μg/m^3^. Examining the yearly trends, 2019 saw the highest average concentrations for NO_2_ (25.5 ± 13.8 μg/m^3^) and O_3_ (48.9 ± 19.5 μg/m^3^), whereas SO_2_ peaked in 2020 (10.8 ± 3.9 μg/m^3^). Figure 3 and Figure 4 visually depict the fluctuations in average air pollutant concentrations across different months and years.

Considering the updated thresholds outlined by the WHO for atmospheric pollutants, NO_2_ exceeded the prescribed 24-hour maximum concentration on 36% of the days. Moreover, during the observed period, O_3_ surpassed this limit in 0.5% of the timeframe, SO_2_ in 0.4%, and PM_10_ in 5.1%. Spearman’s correlation analysis was conducted to assess the relationship between daily pollutant concentrations and meteorological factors detailed in Table 3.

Table 3 illustrates Spearman correlation coefficients examining how daily air pollutant concentrations relate to climatological variables. NO_2_ displayed a moderate positive correlation with PM_10_ (r = 0.33, *p* < 0.01) and a moderate negative correlation with O_3_ (r = −0.31, *p* < 0.01). On the other hand, PM_10_ showed a weak negative correlation with O_3_ (r = −0.17, *p* < 0.01) and a slight positive correlation with SO_2_ (r = 0.16, *p* < 0.01), with no significant correlation with relative humidity (RH) (r = −0.03, *p* > 0.05). O_3_ exhibited a strong negative correlation with RH (r = −0.63, *p* < 0.01), a moderate positive correlation with temperature (r = 0.43, *p* < 0.01), and a weak positive correlation with SO2 (r = 0.11, *p* < 0.01). The observed positive correlation between O_3_ and temperature can be attributed to the heightened photochemical formation of O3 on warm, sunny days. Conversely, the negative correlation between O_3_ and NO_2_ likely arises from the fact that NO_2_ serves as a precursor to the formation of O_3_. In contrast, SO_2_ did not display a significant correlation with NO_2_ (r = −0.01, *p* > 0.05) but demonstrated a weak positive correlation with PM_10_ (r = 0.16, *p* < 0.01) and a negative correlation with meteorological variables (r = −0.08 and −0.05). Overall, these findings indicate varying degrees of association between air pollutants and weather variables.

### 3.3. Evaluating the Influence of Elevated Atmospheric Pollutants Exceeding WHO Limits on Non-ST-Segment Elevation Acute Coronary Syndrome Hospital Admissions

In our investigation, we conducted a multivariable Poisson regression analysis to explore the link between days surpassing WHO-recommended pollutant thresholds and daily NSTE-ACS admissions. This examination was stratified across various factors: season; gender (men vs. women); age (<65 years vs. ≥65 years); CAD (single-vessel lesions vs. multi-vessel lesions); type of NSTE-ACS (NSTEMI vs. UA); and presence or absence of CVRF (DM+ vs. DM−; HTN+ vs. HTN−). Pollutants such as O_3_ and SO_2_, which surpassed the predefined threshold on a limited number of days (0.52% for O_3_ and 0.37% for SO_2_), were also included in the statistical analysis to ensure a more accurate evaluation of the influence of atmospheric pollutants on acute coronary events’ hospitalization rates. Our seasonal breakdown revealed that days with an average NO_2_ concentration ≥25 μg/m^3^ were associated with a 22.3% overall increase in NSTE-ACS admissions (OR: 1.223, 95% CI 1.125–1.330; *p* < 0.001). Specifically, this increase was 42.6% during spring (OR: 1.426, 95% CI 1.196–1.710; *p* < 0.001) and 26.3% in summer (OR: 1.263, 95% CI 1.072–1.487; *p* = 0.005). A concise summary of these findings can be found in Table 4.

The subgroup analysis unveiled a 10.1% rise (OR: 1.101, 95% CI 1.007–1.204; *p* = 0.035) in daily hospitalizations for NSTE-ACS among hypertensive patients when NO_2_ exceeded limit values. Surpassing the NO_2_ threshold elevated the risk of hospitalizations for UA by 10.7% (OR: 1.107, 95% CI 1.010–1.213; *p* = 0.030) compared to instances of NSTEMI. The examination conducted on other subgroups, including gender, age, and CAD, did not yield statistically significant results. Comprehensive details of these findings can be found in Table 5.

### 3.4. Assessing the Influence of Brief Increases in Atmospheric Pollutant Concentrations (≥10 μg/m^3^) on Non-ST-segment Elevation Acute Coronary Syndrome Hospital Admissions

We extended our investigation to assess the impact of short-term increases in atmospheric pollutant concentrations at different lag periods (lag 0–3; lag 0–5; lag 0–7) on the daily count of NSTE-ACS admissions, stratified by seasons. Table 6 provides a comprehensive summary of the multivariable Poisson regression analysis, elucidating the association between each increment of ≥10 μg/m^3^ in environmental pollutants at various time intervals and the occurrence of acute coronary events, categorized by season.

The cumulative impact of NO_2_ revealed a 0.5% rise in the daily count of NSTE-ACS hospitalizations for every 10 μg/m^3^ increase at lag 03 (OR: 1.005, 95% CI 1.001–1.010; *p* = 0.017) and lag 05 (OR: 1.005, 95% CI 1.000–1.009; *p* = 0.041), with a slightly higher association at lag 07 (OR: 1.006, 95% CI 1.001–1.011; *p* = 0.010), indicating a risk increase of 0.6%. Notably, a substantial variation in the impact was observed across different seasons. In the spring period, each 10 μg/m^3^ increase in NO_2_ was associated with a 1.3% rise in NSTE-ACS admissions at lag 03 (OR: 1.013, 95% CI 1.001–1.024; *p* = 0.027) and lag 07 (OR: 1.013, 95% CI 1.001–1.025; *p* = 0.033), while in the summer season, a 1% increase in risk was observed at lag 07 (OR: 1.010, 95% CI 1.001–1.019; *p* = 0.032). Additionally, during winter, each 10 μg/m^3^ rise in O_3_ levels at lag 03 (OR: 1.007, 95% CI 1.001–1.013; *p* = 0.029) and lag 05 (OR: 1.007, 95% CI 1.001–1.014; *p* = 0.025) resulted in a 0.7% increase in daily number of acute coronary events, with a more statistically significant impact at lag 05. No significant association was found regarding SO_2_ and PM_10_ for any lag days, possibly explained by the lower levels of these pollutants in our country.

These findings shed light on the detrimental effects of increased environmental pollutant emissions on human well-being. It underscores the urgency of implementing robust measures to curb air pollution across energy, industry, agriculture, and transportation sectors, as well as within the healthcare domain. The primary aim is to mitigate risks posed to both environmental and human health.

## 4. Discussion

This prospective observational study, to the best of our knowledge, marks the first investigation conducted in Eastern Europe, providing insights into the correlation between short-term exposure to air pollutants and the daily hospitalization rates for NSTE-ACS. Our research findings unveil a notable association between elevated NO_2_ concentrations surpassing WHO reference limits at lag 01 days and a 22.3% increase in daily NSTE-ACS occurrences, regardless of the season. Specifically, during spring, surpassing the NO_2_ reference level correlates with a substantial 42.6% surge in NSTE-ACS incidence. Moreover, heightened NO_2_ concentrations during summer are linked to a 26.3% increase in daily admissions for acute coronary events.

The subgroup analysis revealed a 10.7% increase in cases of UA alongside a 10.1% heightened risk of acute coronary disease among hypertensive patients on days with elevated NO_2_ levels. Milojevic et al. identified an increased risk of NSTEMI associated with exposure to heightened NO_2_ concentrations at lags 0–4 days [30]. Another epidemiological study conducted in France reported an escalated risk of acute coronary events when NO_2_ concentrations exceeded 30 μg/m^3^ [17]. Canadian study data indicated a raised risk of NSTEMI among elderly and hypertensive patients exposed to elevated NO_2_ levels [31]. A study conducted in Poland demonstrated a 9% increase in the risk of NSTEMI and an 11% increase in the risk of UA on days with heightened NO_2_ concentrations [32].

In our study, we noted a progressive increase in daily NSTE-ACS admissions, ranging from 0.5% at lag 03 to 0.6% at lag 07, for each 10 μg/m^3^ rise in NO_2_ concentration. Differing impacts were noted across seasons, where in the spring, a 10 μg/m^3^ increase in NO_2_ was linked to a 1.3% rise in NSTE-ACS admissions at lag 03 and 07, whereas a slightly lower risk of 1% in admissions was observed during the summer season at lag 07. Corresponding with our findings, a systematic review indicated a 1.1% increase in the risk of acute myocardial infarction (AMI) for every 10 μg/m^3^ rise in NO_2_ at lag 1 [33]. Results from a study in Poland demonstrated an increase in NSTEMI hospitalizations, both in industrial areas (by 6.2%) and non-industrial areas (by 12.6%), with each 10 μg/m^3^ rise in NO_2_ [34]. Sahlén et al. reported a 4.2% increase in the risk of STEMI for each 12.9 μg/m^3^ increase in NO_2_, while Butland et al. found a 10 μg/m^3^ rise in NO_2_ levels to be associated with a 0.27% increase in the risk of NSTEMI [35,36].

The results from our analysis, evaluating the influence of elevated PM_10_ and SO_2_ concentrations surpassing WHO-recommended thresholds at lag 01 on NSTE-ACS hospitalizations, did not produce statistically significant findings. This could be attributed to the limited surpassing of daily PM_10_ and SO_2_ concentration norms, with only 5.1% and 0.37%, respectively, during the study period. No significant associations were observed for SO_2_ and PM_10_ across any lag days, potentially explained by the lower levels of these pollutants in our country. PM_2.5_ was not included in the analysis due to a considerable number of missing values within the study timeframe.

The epidemiological evidence has substantiated the adverse impact of PM_10_ on cardiovascular health, showcasing an increased occurrence of UA cases in elderly patients following a 10 μg/m^3^ rise in PM_10_ concentration [19]. Findings from an Italian study indicated a 1.1% elevation in ACS risk for every 14 μg/m^3^ increase in PM_10_ and a 2.3% increase for each 10 μg/m^3^ rise in PM_2.5_ [18]. Moreover, Cesaroni et al. demonstrated a 12% and 13% escalation, respectively, in the risk of acute coronary diseases due to chronic exposure to PM_10_ and PM_2.5_ [37]. The scientific literature consistently highlights a more detrimental impact of PM_2.5_ compared to PM_10_ on human health [20,33], attributed to its larger surface area facilitating increased absorption of chemical constituents and its extended persistence in ambient air, enhancing its capability to reach pulmonary alveoli [20,38].

In the winter season, there was a 0.7% rise in daily NSTE-ACS hospitalizations associated with a 10 μg/m^3^ increase in O_3_ concentration at lag 03 and 05, with a more notable statistical significance observed at lag 05. Correspondingly, a study conducted in China exhibited a 1.3% increase for every 10 μg/m^3^ rise in the average O_3_ concentration at lag 05 [39]. Additionally, a recent investigation indicated a 0.75% increase in AMI hospitalizations for each 10 μg/m^3^ elevation in daily 8-hour maximum O_3_ concentration at lag 01. Furthermore, surpassing the O_3_ level recommended by WHO guidelines led to a 6.52% escalation in the risk of acute cardiovascular events [12]. Ruidavets et al. also observed an augmented risk of AMI due to exposure to ambient O_3_ over a 1 to 2-day period [11]. Nevertheless, some studies have found no association between O_3_ exposure and the daily count of hospitalizations for cardiovascular diseases [40,41].

The literature encompasses a multitude of pathophysiological mechanisms elucidating the onset of ACS. 1. The main cause is coronary thrombosis, which often involves rupture of the protective fibrous cap surrounding the atherosclerotic plaque; 2. Another mechanism involves an altered equilibrium between the prothrombotic and fibrinolytic properties of the endothelium, potentially leading to thrombosis in situ; 3. In a minority of instances, a fatal thrombosis within coronary arteries may occur due to a superficial erosion of the intima, lacking a clear rupture through the fibrous cap of the plaque. Inflammation is also recognized as a potential contributor to this coronary thrombosis mechanism; 4. Vasospasm, a significant pathophysiological mechanism, can further contribute to compromised arterial flow in the presence of inflammation. Endothelial cells in atherosclerotic arteries exhibit impaired vasodilator function, partly attributed to reduced nitric oxide production, which possesses the ability to hinder platelet aggregation and concurrently exerts a direct anti-inflammatory effect [7]. The precise pathophysiological mechanism by which exposure to elevated ambient air pollutant concentrations may contribute to acute coronary events remains incompletely understood. Previous studies have suggested that NO_2_ could induce proinflammatory and prothrombotic effects, leading to endothelial dysfunction, thus potentially facilitating the onset of AMI [23,26]. Researchers have proposed several potential mechanisms through which exposure to PM_10_ and PM_2.5_ could trigger AMI, including their association with prothrombotic and proinflammatory states, as well as the induction of endothelial dysfunction [13,20,42]. Notably, a recent clinical study revealed that ACS patients exposed to elevated concentrations of PM_2.5_ exhibited a ruptured atheroma plaque as the mechanism underlying the culprit injury, observed via optical coherence tomography (OCT) [43]. Similarly to various atmospheric pollutants, clinical investigations have highlighted O_3_’s capacity to induce fibrinolysis, promote a prothrombotic state, and cause endothelial dysfunction [24,44]. Moreover, as an exceptionally reactive pollutant, O_3_ can initiate the oxidation of cholesterol within atheroma plaques, potentially fostering the development of unstable plaque [45]. Therefore, short-term exposure to environmental pollutants increases the risk of hospitalization for NSTE-ACS, mainly due to the destabilization of atherosclerotic plaques caused by oxidative stress, inflammation, and endothelial dysfunction.

The present findings provide valuable insights into the adverse consequences arising from heightened emissions of environmental pollutants in acute coronary events. It is imperative to implement impactful measures aimed at curbing air pollution across diverse sectors, including energy, industry, transport, agriculture, and healthcare, with the overarching objective of reducing associated health risks.

## 5. Conclusions

The findings underscore the nuanced association between environmental pollutants and the likelihood of NSTE-ACS hospitalizations, especially pronounced in specific seasons. Elevated risks of hospital admissions for acute coronary events were identified on days with high NO_2_ levels, notably during the spring season. Hypertensive individuals and those diagnosed with UA exhibited heightened susceptibility to elevated NO_2_ concentrations. Each 10 μg/m^3^ increase in NO_2_ in spring and O_3_ in winter was linked to higher NSTE-ACS admissions, with a more pronounced effect at lag 07 for NO_2_ and lag 05 for O_3_, respectively. The external validity of our findings is restricted to areas with similar environmental features and prevalence of acute coronary disease. Hence, prudence is advised in interpreting the outcomes, and additional research is necessary. It is crucial in contemporary contexts to broaden the focus beyond traditional cardiovascular risk factors and include emerging environmental risk factors, acknowledging their considerable impact on both human health and the environment.

### Study Limitations

This study is subject to certain limitations. It focused exclusively on the relationship between outdoor air pollution and daily hospitalization rates for NSTE-ACS (encompassing NSTEMI and UA) while excluding patients with STEMI, CCS, or other cardiovascular conditions. This limitation implies that this study may not fully represent the broader spectrum of cardiovascular diseases influenced by air pollution. Factors like pre-existing health conditions, medication usage, and socio-economic status, which could impact susceptibility to ambient air pollution, were not captured due to the unavailability of relevant data. The study sample was derived from a single university center, and pollutant measurements were obtained from fixed monitoring stations near participants’ residences, limiting the assessment of individual exposure to indoor air pollution. Additionally, due to substantial periods of missing data or lack of measurements, this study did not include an analysis of substances like PM_2.5_ and ultrafine particulates. This absence might lead to underestimating the potential health impacts of air pollution.

## Figures and Tables

**Table 1:** Descriptive statistics of daily non-ST-segment elevation acute coronary syndrome admissions.

	NSTE-ACS (*n* = 1547)
**Age** (years)	63.53 ± 10.0
**Gender** (male) (female)	1122 (72.5%) 425 (27.5%)
**BMI** (kg/m^2^)	29.23 ± 5.1
**Clinical and paraclinical parameters at admission**	
SBP (mmHg)	138.95 ± 21.4
DBP (mmHg)	80.18 ± 12.0
HR (bpm)	72.52 ± 14.1
Hemoglobin (mg/dL)	13.96 ± 1.8
Creatinine (mg/dL)	1.19 ± 0.8
Glucose (mg/dL)	129.68 ± 52.6
Total cholesterol (mg/dL)	153.07 ± 44.8
Triglycerides (mg/dL)	151.43 ± 91.5
LDL-cholesterol (mg/dL)	94.08 ± 33.6
HDL-cholesterol (mg/dL)	34.756 ± 15.6
**Cardiovascular risk factors**	
Smoking	354 (22.9%)
Arterial hypertension	1341 (86.7%)
Diabetes mellitus	506 (32.7%)
Hypercholesterolemia	588 (38%)
**Coronary arteries lesions**	
Single-vessel lesion	436 (28.2%)
Two-vessel lesion	542 (35%)
Three-vessel lesion	569 (36.8%)
**Treatment at admission**	
PCI primary	1306 (84.4%)
Balloon angioplasty	19 (1.2%)
CABG	88 (5.7%)
Conservative	134 (8.7%)
**Type of NSTE-ACS**	
NSTEMI	264 (17.1%)
UA	1283 (82.9%)

Values are mean ± SD or number (%). NSTE-ACS: Non-ST-segment Elevation Acute Coronary Syndrome; BMI: Body Mass Index; SBP: Systolic Blood Pressure; DBP: Diastolic Blood Pressure; HR: Heart Rate; PCI: Percutaneous Coronary Intervention; CABG: Coronary Artery Bypass Grafting; NSTEMI: Non-ST-segment Elevation Myocardial Infarction; UA: Unstable Angina.

**Table 2:** Descriptive statistics of air pollutants and meteorological variables.

Total Days (*n* = 1908)	Mean ± SD	Minimum	Percentiles			Maximum
			25th	50th	75th	
**NO_2_ (μg/m^3^)**	23.0 ± 11.7	0.5	14.6	21.0	29.3	81.2
**O_3_ (μg/m^3^)**	43.9 ± 19.3	4.3	28.7	43.7	56.9	141.7
**SO_2_ (μg/m^3^)**	10.2 ± 3.6	3.7	8.0	9.3	11.2	43.3
**PM_10_ (μg/m^3^)**	21.2 ± 13.6	1.5	12.3	18.2	26.3	114.0
**Air temperature (°C)**	12.7 ± 8.5	−8.3	5.7	12.5	19.7	30.4
**Relative humidity (%)**	72.4 ± 14.2	32.0	62	73	84	100.0

The total of 1908 data days does not correspond to the total calendar days over the 3-year period due to data collection from 5 regions, each with specific variations in daily air pollution and meteorological parameters. NO_2_: nitrogen dioxide; O_3_: ozone; SO_2_: sulfur dioxide; PM_10_: particulate matter with a diameter ≤10 μm.

**Table 3:** Spearman’s correlation coefficients evaluate the relationship between daily air pollutant concentrations and climatological variables.

	NO_2_	O_3_	SO_2_	PM_10_	Temperature	Relative Humidity
**NO_2_**	1	−0.31 **	−0.01	0.33 **	−0.14 **	0.00
**O_3_**	−0.31 **	1	0.11 **	−0.17 **	0.43 **	−0.63 **
**SO_2_**	−0.01	0.11 **	1	0.16 **	−0.08 **	−0.05 *
**PM_10_**	0.33 **	−0.17 **	0.16 **	1	−0.08 **	−0.03
**Temperature**	−0.14 **	0.43 **	−0.08 **	−0.08 **	1	−0.51 **
**Relative** **humidity**	0.00	−0.63 **	−0.05 *	−0.03	−0.51 **	1

The Spearman correlation analysis involves 15 pairs of variables. * *p* < 0.05; ** *p* < 0.01; NO_2_: nitrogen dioxide; O_3_: ozone; SO_2_: sulfur dioxide; PM_10_: particulate matter with a diameter ≤10 μm.

**Table 4:** Multivariable Poisson regression analysis: the relationship between days surpassing pollutant concentration thresholds and daily incidence of non-ST-segment elevation acute coronary syndrome at lag 01, stratified by season.

Air Pollutants	NO_2_ ≥ 25 μg/m^3^ (WHO Guideline)		O_3_ ≥ 100 μg/m^3^ (WHO Guideline) *		SO_2_ ≥ 40 μg/m^3^ (WHO Guideline) *		PM_10_ ≥ 45 μg/m^3^ (WHO Guideline)	
	Odds Ratio (95% CI)	*p*-Value	Odds Ratio (95% CI)	*p*-Value	Odds Ratio (95% CI)	*p*-Value	Odds Ratio (95% CI)	*p*-Value
Season								
All Seasons	1.223 (1.125–1.330)	**<0.001**	0.652 (0.308–1.379)	0.263	0.689 (0.172–2.759)	0.598	0.896 (0.742–1.082)	0.252
3 Winters	1.077 (0.909–1.278)	0.391	0.699 (0.097–5.036)	0.722	0.843 (0.208–3.408)	0.811	0.840 (0.606–1.163)	0.293
3 Springs	1.426 (1.196–1.701)	**<0.001**	0.728 (0.321–1.649)	0.446	-	-	0.884 (0.470–1.664)	0.703
3 Summers	1.263 (1.072–1.487)	**0.005**	-	-	-	-	1.274 (0.673–2.411)	0.458
3 Falls	1.143 (0.968–1.350)	0.115	-	-	-	-	0.955 (0.718–1.272)	0.754

The total of 1908 data days does not correspond to the total calendar days over the 3-year period due to data collection from 5 regions, each with specific variations in daily air pollution levels. * Pollutants such as O_3_ and SO_2_, which surpassed the predefined threshold on a limited number of days (0.52% for O_3_ and 0.37% for SO_2_), were also included in the statistical analysis to ensure a more accurate evaluation of the influence of atmospheric pollutants on acute coronary events’ hospitalization rates. The utilization of the “-” symbol does not imply any error or shortcoming in the analysis; rather, it signifies that the instances of exceeding the threshold for this pollutant were scarce. “Lag 01” represents the moving average of the current day and the previous day’s exposure to air pollutants. All WHO reference values for air pollutants are presented on a 24-hour basis, except the 8-hour maximum daily average concentration for O_3_. An observed OR exceeding 1 suggests an increased risk, while a value below 1 indicates a reduced risk. A *p*-value below 0.05 signifies a statistically significant association. Seasons were defined as winter (December–February), spring (March–May), summer (June–August), and fall (September–November). NO_2_: nitrogen dioxide; PM_10_: particulate matter with a diameter ≤10 μm; O_3_: ozone; SO_2_: sulfur dioxide; WHO: World Health Organization; CI: confidence interval; OR: odds ratio.

**Table 5:** Multivariable Poisson regression analysis: daily incidence of non-ST-segment elevation acute coronary syndrome in relation to exceeded pollutant concentration thresholds at lag 01, stratified by various subgroups.

	NO_2_ ≥ 25 μg/m^3^ (WHO Guideline)		O_3_ ≥ 100 μg/m^3^ (WHO Guideline) *		SO_2_ ≥ 40 μg/m^3^ (WHO Guideline) *		PM_10_ ≥ 45 μg/m^3^ (WHO Guideline)	
Subgroups	Odds Ratio (95% CI)	*p*-Value	Odds Ratio (95% CI)	*p*-Value	Odds Ratio (95% CI)	*p*-Value	Odds Ratio (95% CI)	*p*-Value
**Gender**								
Male (*n* = 1122)	1.034 (0.934–1.143)	0.521	0.676 (0.251–1.816)	0.437	0.628 (0.157–2.519)	0.512	0.853 (0.660–1.103)	0.226
Female (*n* = 425)	1.114 (0.951–1.304)	0.181	0.629 (0.154–2.572)	0.519	-	-	0.942 (0.655–1.355)	0.747
**Age**								
<65 (*n* = 797)	1.095 (0.972–1.234)	0.134	0.655 (0.208–2.058)	0.468	0.610 (0.152–2.448)	0.486	0.873 (0.656–1.160)	0.349
≥65 (*n* = 750)	1.024 (0.907–1.156)	0.700	0.667 (0.213–2.092)	0.488	-	-	0.889 (0.652–1.211)	0.456
**Patients** **with/without CVRF**								
DM + (*n* = 506)	0.998 (0.858–1.162)	0.984	0.660 (0.242–1.800)	0.417	0.609 (0.085–4.349)	0.621	0.935 (0.641–1.363)	0.726
DM − (*n* = 1041)	1.089 (0.982–1.208)	0.105	0.667 (0.166–2.685)	0.569	0.609 (0.086–4.334)	0.621	0.859 (0.667–1.106)	0.237
HTN + (*n* = 1341)	1.101 (1.007–1.204)	**0.035**	0.660 (0.311–1.398)	0.278	0.595 (0.148–2.383)	0.463	0.858 (0.697–1.057)	0.150
HTN − (*n* = 206)	0.982 (0.757–1.272)	0.888	-	-	-	-	1.255 (0.791–1.992)	0.334
**Coronary artery** **disease**								
Single-vessel lesions (*n* = 436)	1.009 (0.853–1.192)	0.918	0.707 (0.224–2.232)	0.554	-	-	0.880 (0.558–1.390)	0.585
Multi-vessel lesions (*n* = 1111)	1.072 (0.971–1.183)	0.167	0.643 (0.205–2.016)	0.449	0.596 (0.149–2.387)	0.464	0.878 (0.693–1.112)	0.279
**Type of NSTE-ACS**								
NSTEMI (*n* = 1283)	0.997 (0.810–1.227)	0.975	0.603 (0.258–1.412)	0.244	-	-	0.829 (0.489–1.408)	0.488
UA (*n* = 264)	1.107 (1.010–1.213)	**0.030**	0.673 (0.095–4.792)	0.693	0.602 (0.150–2.413)	0.474	0.915 (0.748–1.120)	0.390

* Pollutants such as O_3_ and SO_2_, which surpassed the predefined threshold on a limited number of days (0.52% for O_3_ and 0.37% for SO_2_), were also included in the statistical analysis to ensure a more accurate evaluation of the influence of atmospheric pollutants on acute coronary events’ hospitalization rates. The utilization of the “-” symbol does not imply any error or shortcoming in the analysis; rather, it signifies that the instances of exceeding the threshold for this pollutant were scarce. “Lag 01” represents the moving average of the current day and the previous day’s exposure to air pollutants. All WHO reference values for air pollutants are presented on a 24-h basis, except the 8-hour maximum daily average concentration for O_3_. An observed OR exceeding 1 suggests an increased risk, while a value below 1 indicates a reduced risk. A *p*-value below 0.05 signifies a statistically significant association. NO_2_: nitrogen dioxide; PM_10_: particulate matter with a diameter ≤ 10μm; O_3_: ozone; SO_2_: sulfur dioxide; WHO: World Health Organization; CI: confidence interval; OR: odds ratio; CVRF: cardiovascular risk factors; DM: diabetes mellitus; HTN: hypertension; NSTE-ACS: non-ST-segment elevation acute coronary syndrome; NSTEMI: non-ST-segment elevation myocardial infarction; UA: unstable angina.

**Table 6:** Multivariable Poisson regression analysis: impact of each ≥10 μg/m^3^ increase in pollutant concentrations at different time lags on daily non-ST-segment elevation acute coronary syndrome incidence, stratified by seasons.

	NO_2_ (μg/m^3^)		O_3_ (μg/m^3^)		SO_2_ (μg/m^3^)		PM_10_ (μg/m^3^)	
	Odds Ratio (95% CI)	*p*-Value	Odds Ratio (95% CI)	*p*-Value	Odds Ratio (95% CI)	*p*-Value	Odds Ratio (95% CI)	*p*-Value
**Lag 03**								
All Season	1.005 (1.001–1.010)	**0.017**	1.002 (0.998–1.005)	0.288	0.987 (0.973–1.002)	0.083	0.998 (0.994–1.001)	0.213
3 Winters	1.005 (0.997–1.014)	0.179	1.007 (1.001–1.013)	**0.029**	0.996 (0.971–1.022)	0.770	0.994 (0.987–1.002)	0.151
3 Springs	1.013 (1.001–1.024)	**0.027**	1.004 (0.996–1.011)	0.309	0.972 (0.936–1.009)	0.132	0.994 (0.982–1.006)	0.301
3 Summers	1.007 (0.999–1.016)	0.085	1.002 (0.994–1.010)	0.603	0.976 (0.949–1.004)	0.095	0.998 (0.986–1.011)	0.793
3 Falls	0.997 (0.986–1.007)	0.543	0.996 (0.988–1.055)	0.424	0.998 (0.968–1.030)	0.923	1.001 (0.993–1.008)	0.881
**Lag 05**								
All Season	1.005 (1.000–1.009)	**0.041**	1.002 (0.999–1.006)	0.220	0.983 (0.968–0.998)	0.310	0.998 (0.994–1.002)	0.333
3 Winters	1.005 (0.997–1.013)	0.258	1.007 (1.001–1.014)	**0.025**	0.995 (0.969–1.022)	0.717	0.994 (0.986–1.003)	0.180
3 Springs	1.010 (0.998–1.022)	0.088	1.004 (0.996–1.011)	0.365	0.958 (0.920–0.996)	0.321	0.999 (0.986–1.012)	0.853
3 Summers	1.008 (0.999–1.017)	0.090	1.002 (0.993–1.011)	0.618	0.978 (0.951–1.006)	0.130	1.001 (0.988–1.015)	0.852
3 Falls	0.996 (0.985–1.007)	0.437	0.997 (0.988–1.006)	0.524	0.991 (0.960–1.024)	0.598	1.001 (0.993–1.009)	0.795
**Lag 07**								
All Season	1.006 (1.001–1.011)	**0.010**	1.002 (0.998–1.005)	0.364	0.983 (0.968–0.999)	0.351	0.998 (0.994–1.002)	0.384
3 Winters	1.004 (0.996–1.013)	0.300	1.006 (1.000–1.013)	0.067	0.996 (0.970–1.023)	0.764	0.996 (0.987–1.004)	0.302
3 Springs	1.013 (1.001–1.025)	**0.033**	1.004 (0.996–1.012)	0.346	0.950 (0.912–0.989)	0.130	1.006 (0.992–1.020)	0.386
3 Summers	1.010 (1.001–1.019)	**0.032**	1.002 (0.992–1.011)	0.745	0.984 (0.957–1.012)	0.255	1.004 (0.991–1.018)	0.538
3 Falls	0.997 (0.986–1.008)	0.579	0.995 (0.986–1.005)	0.317	0.993 (0.961–1.026)	0.664	0.999 (0.991–1.007)	0.812

The total of 1908 data days does not correspond to the total calendar days over the 3-year period due to data collection from 5 regions, each with specific variations in daily air pollution levels. An observed OR exceeding 1 suggests an increased risk, while a value below 1 indicates a reduced risk. A *p*-value below 0.05 signifies a statistically significant association. The terms “lag 03”, “lag 05”, and “lag 07” denote the moving averages of air pollutant concentrations from the preceding three, five, and seven days, respectively. Seasons were defined as winter (December–February), spring (March–May), summer (June–August), and fall (September-November). NO_2_: nitrogen dioxide; O_3_: ozone; PM_10_: particulate matter with a diameter ≤10 μm; CI: confidence interval; OR: odds ratio.

## Data Availability

The data underlying this article is not publicly available to ensure the privacy of the study participants. However, interested parties may request access to the data by contacting the corresponding author.
